# A comprehensive study on the fire resistance properties of ultra-fine ceramic waste-filled high alkaline white cement paste composites for progressing towards sustainability

**DOI:** 10.1038/s41598-023-49229-4

**Published:** 2023-12-13

**Authors:** M. A. Abdelzaher, Asmaa S. Hamouda, Ibrahim M. El-Kattan

**Affiliations:** Environmental Science and Industrial Development Department, Faculty of Postgraduate Studies for Advanced Sciences, Beni-Suef University, Beni-Suef, 62511 Egypt

**Keywords:** Ceramics, Composites, Environmental impact

## Abstract

The most practical sustainable development options to safeguard the local ecology involve reducing the use of raw materials and guaranteeing proper recycling of the principal destroyed solid wastes. Preventing the creation of hazardous waste and the subsequent pollution that results from improper disposal is a top priority. Based on this, the study's authors recommend reusing the ultra-fine ceramic shards (CW). High-alkaline white cement (WC) has been partially replaced by ultra-fine CW because it is a cheaper, more abundant, and more lasting environmental material used in the production of trendy blended white cement pastes composites. In this context, we look at ultra-fine CW, a material that has been suggested for use as a hydraulic filler due to its high performance, physicomechanical qualities, and durability. XRF, XRD, FTIR, and SEM measurements are used to characterize the microstructure, thermal characteristics, and thermodynamics. Because of the effect of ultra-fine ceramic waste, the firing test reduces the mechanical strength by default, but with active filler, decreases slowly and increase its physicomechanical features and compressive strength compared to the control sample (WC), setting a new benchmark. The maximum amount of crystallization formed in the presence of ultra-fine ceramic waste in WC-matrix, resulting in a decrease in total porosity and early cracking. Together, the improved workability and energy-saving features of cement blends with ultra-fine ceramic waste, reflect their economic and environmental benefits, which may reduce building costs and boost the durability of the raw materials used in the mix.

## Introduction

Effective sustainable strategies for more sustainable resource usage cannot be developed without a solid understanding of community consumption, and/or developing local population bias towards waste optimization for continuous economic growth and circular economy. Rapid urbanization in recent decades has fueled the growth of the construction sector, but this boom has come at the expense of the environment through the wasteful use of energy and materials. The results of these actions are extremely detrimental to ecosystems and human health^1,2^.

The widespread adoption of sustainable building materials has been seen as one of the most promising approaches to boost the building sector's performance in a sustainable way. Sustainable concepts have been included into the design, construction, decorating, operation, and maintenance of buildings to lessen the negative impact on the environment, natural resources, the health, and the comfort of the people. Sustainable construction material selection is widely seen as the quickest and most important technique to achieve sustainability out of all these initiatives^3–5^.

There is an increase in the amount of solid trash in the surrounding environment, but very little potential for its reuse or recycling. Unplanned consumption of natural resources can be slowed by using modern waste management technologies; nonetheless, MSW; has significant detrimental effects on both built environment and human health. As production volumes rise, there is also an increase in the proportion of trash that is solid, which has an adverse effect on the environment. Many national and international regulations stress the need of researching garbage recycling in an effort to lessen its harmful effects. In order to achieve greater eco-efficiency and to find more applicable materials, several investigations have concentrated on marble/ceramic wastes^6,7^.

The rising demand for eco-friendly, low-cost, and hazard-free building materials has prompted researchers to dig into the question of how this can be done on a large scale while still meeting the environmental and material needs affirmed by acceptable limits^2,4,8^. Ceramics have become increasingly popular in the building industry, especially in recent years. So, when ceramic industries crank out more goods, there will not be enough space to stash all the resulting ceramic waste (CW). Potential annual ceramic waste production in 2025 is estimated at 25 BT^9^. Reduce environmental damage and boost the economy with this innovative approach to dealing with industrial trash^10–13^. Inorganic materials, that are used as binders in construction industry e.g.: SCMs, has effective operation nowadays in multiple advanced industries such as carton, vertical agro, plastics, organic composite, glass and concrete.

One of those material which is a byproduct from industries, ceramic waste (CW) may be seen as effective natural source as; it slow cost and hydraulic features^14,15^. One of the main applications of cement is the manufacture of concrete, which requires the use of aggregates that can significantly alter fire resistance.

The economic aspects, environmental impacts, and priorities of renewable resources are main factories, which detected the principal of recycling application of both natural and inorganic materials. The main purpose of renewable and alternatives inorganic fillers is their reuse as SCMs. These fillers are characterized with low cost, high efficiency, high mechanical properties and durability^16,17^. Additionally, a review of the literature by Arel^18^ has tested several studies focusing on the applications of ceramic wastes as replacement in concrete aggregates.

The recycling of ceramic wastes instead of aggregate into concrete has enhanced compressive mechanical strength and durability. The measurements indicated, the substation of ceramic wastes instead of aggregate enhanced workability as it reduces water consistency^19^. Meanwhile, the replacement of cement/concrete by waste ceramics powder reduce in CO_2_ gas emissions by 12% and reducing fixed cost up to $39/m^3^^20^. Recently; a trendy track, known as “paste replacement method” was proposed by certain researchers. According to this method, ceramic waste is subsided from the cement mix content without any change in the W/C ratio.

This method proved that; the durability of the cement motor was significantly enhanced by a mix content of around ~ 33%^21^. Previous research demonstrated that ~ 30 to 40% of ceramic production is converted into waste^22^. Cement manufacturing is characterized by consuming huge amounts of material resources and energy in compared with other large industries, which process a possible industrial process to consume numerous industrial wastes decreasing its generation rate^23^. However, raw material prices and growing demand pose a major challenge in the development of the construction field. Egypt, being one of the largest solid waste economies, produces a quantitative volume of solid waste that dumping is obligatory.

Coarse aggregates play an important role as a fiber filler in the production of conventional cement and concrete. The preliminary results of using ceramics waste (WC) in cement production especially succeeded testing of the compressive mechanical strength, durability and setting time behavior^24^. Carbon dioxide and other air pollutants were released into the atmosphere at a rate of 0.44 tons per ton of clinker/cement during manufacturing. More than eight percent of annual global emissions are attributed to cement production, according to some estimates^25^. Cement paste hydration is thought to involve multiple processes, both physical and chemical^26^.

Calcium silicate hydrate (C–S–H) gel's origin and development were extensively discussed by two methods. Topo-chemical theory is the initial mechanism, and reactions by solution are the second once cement begins hydration^27^. White cement production is the largest and most resource-intensive in the manufacturing sector^28^. High raw material use must be somewhat offset before sustainability can be achieved. Cost and carbon dioxide (CO_2_) emission reductions in the manufacturing of white cement are a priority for stakeholders and policymakers^29^. In response to this pressing problem, the research team developed a novel approach to recycling ceramic waste for use as additives in the production of white cement.

The main objective of the current study is to investigate the fire resistance by physico-mechanical properties for mixtures of white cement pastes with ceramic waste. These mixtures have been cured for 28 days and subjected to temperatures from 250 to 750 °C. Cement pastes containing ceramic waste were fired at temperatures ranging from 250 to 750 °C, and their physicomechanical properties were analyzed over a period of 28 days. Physical, chemical, and mechanical properties all pointed to satisfactory development.

The overall results showed that the compressive strength decreased with increasing CW substitution ratios. Therefore, this is the first trial to recycle ceramic waste in its natural state as an alternative material in cement manufacturing, with no additional cost for grinding or treatment process. The group is working on a hands-on program to assess the combined physicochemical and mechanical properties of CW as a partial cement substitute.

## Laboratory program

### Materials

**Figure 1:**
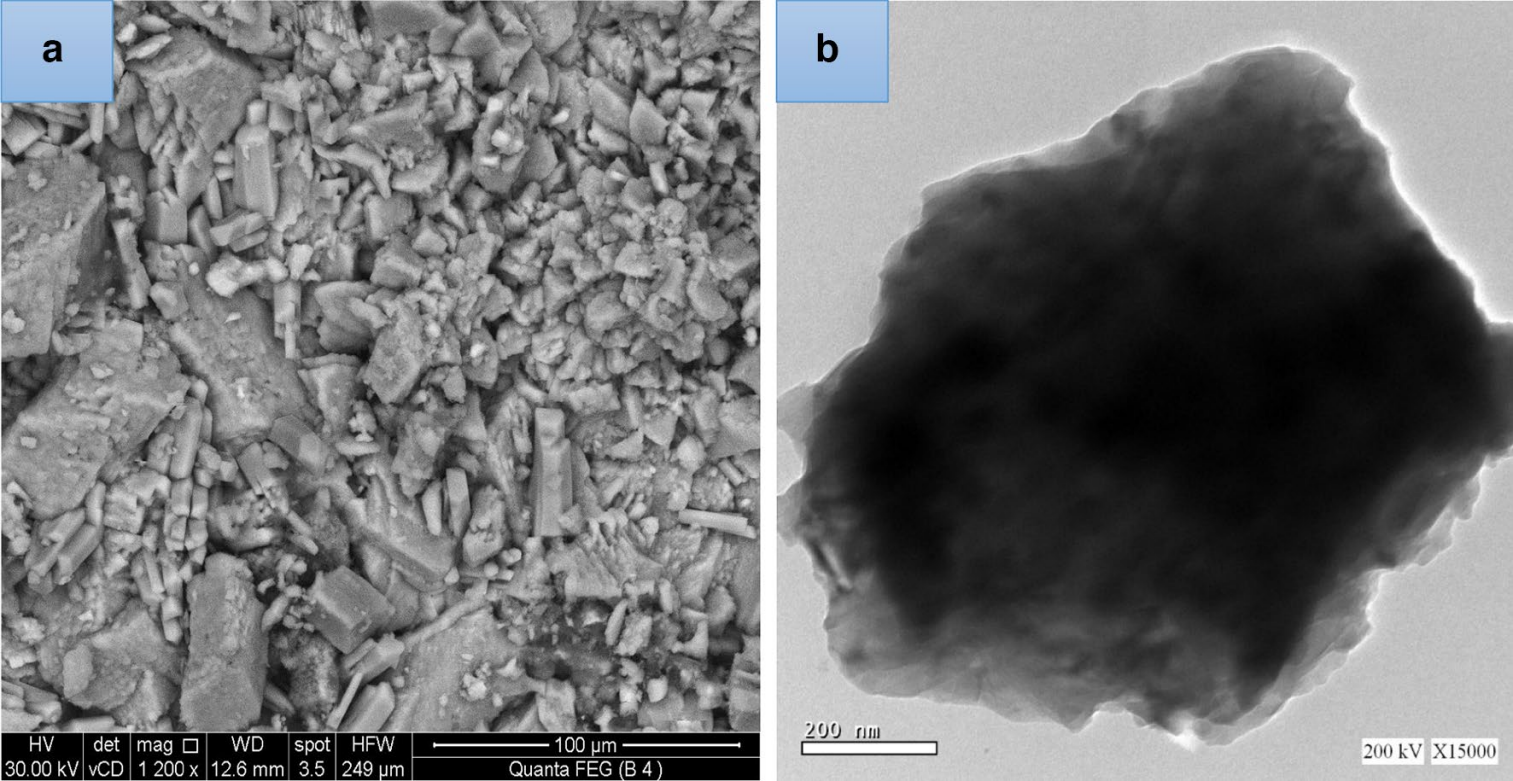
(**a**,**b**) SEM and TEM morphology for ultra-fine CW.

The primary components used in this experimental protocol are ultra-fine ceramic waste (CW) and high-alkaline white cement (WC). CW came from El-Gawhara for ceramic and porcelane company (GCPCo), El-Saddat (Monfita, Egypt), and white ordinary cement (grades I, 52.5 R) came from Saini Cement, Cementier Holding Cement Company (CHCo.), Saini, Egypt. Table 1 displays the results of a comprehensive X-ray fluorescence (XRF) study of the raw materials. According to the bag of cement and confirmed by XRF analysis, the sodium equivalent of high-alkaline white cement is less than 0.6%. Ball milling the CW for 6 h yielded enough fine powder for micro-scaling. The particles sieved through a 45 µ sieve mesh. In addition to its physical and mechanical features of ultra-fine CW, are indicated in Table 2, the specific surface area determined by the fineness test, according to ASTM, C430-08^30^, was 4815 cm^−2^ g. The SEM and TEM images of CW were displayed in Fig. 1a,b. This study revealed that CW particles were spherical in shape, with sizes between 100 and 200 nm.

**Table 1:** XRF analysis of precursors.

Precursors	SiO_2_	Al_2_O_3_	CaO	Fe_2_O_3_	MgO	SO_3_	Na_2_O	K_2_O	LOI	Cl-	P_2_O_5_	Tio_2_
WC	22.48	3.00	68.41	0.14	0.31	2.84	0.23	0.16	2.35	0.08	Nil	Nil
CW	68.02	24.22	0.63	1.92	0.10	Nil	1.65	2.82	0.42	Nil	0.17	0.05

**Table 2:** CW features.

Physicals and mechanicals properties	CW
Grading Index	2.95
Max. partial size (_µ_m)	66.00
Dry density (kg/dm^3^)	2.25
Water consistency	0.49
Porosity (MIP)	0.30

### Preparation and testing methods

Generally, WC was substituted with various potions e.g.: (2.5%, 5%, 10%, 15% and 20) by wt% CW, as reported inside Table 3. Consistency (W/CP) ratios were constant for composites CW.

**Table 3:** The composites pastes %. (As replacement).

Mix composition	White cement batch %	Ultra-fine ceramic waste batch %	Water/cement ratio %	Curing water
M0	100.00	0.00	0.40	Tab water
M2.5F	97.50	2.50	0.40	
M5F	95.00	5.00	0.40	
M10F	90.00	10.00	0.40	
M15F	85.00	15.00	0.40	
M20F	80.00	20.00	0.40	

**Figure 2:**
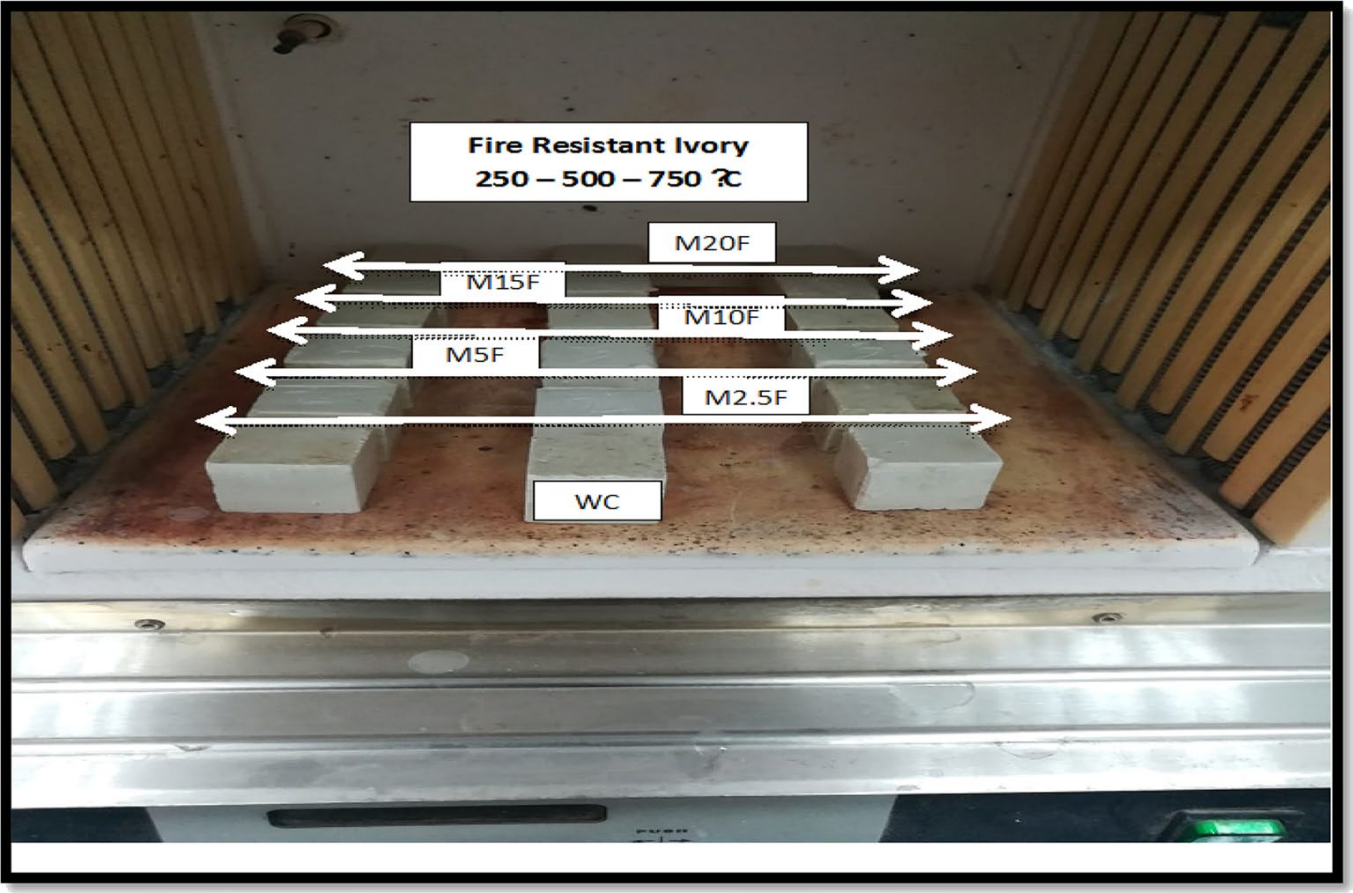
Visual inspection during the synergistic process.

Mixing followed by cast using stainless steel molds with dimensions; (25 mm × 25 mm × 25 mm), start the hydration in 99 ± 1 percentage humidity (RH) at 23 °C. After 24 hs, prisms were stiffening and immersed directly in tap fresh water for up to 28 days of continues hydration then thermally treated at varied temperature scales (250 °C, 500° C and 750 °C) as shown visually during synergistic process in Fig. 2.

Our experimental study, described in greater detail elsewhere^31^, followed the ASTM standard, which highlights the significance of RH; on the hydration kinetics of the specimens. Estimates were made of the changes in compressive strength, porosity, and whiteness reflection that occur in white cement pastes under a wide range of high temperature conditions. Triplicate tests were run on the material's Compressive Strength (CS) in accordance with ASTM C109M^32,33^, with weight 5.00 tons and rating 20 kg per minute by (Shemizitu Machine test) with a loading rate of 25 kg/min. Reflectance measurements of whiteness (Ry) were taken using Elerpho French equipment in accordance with DIN 5033 standards^34^. Mercury intrusion data was used to complete the solidification of the prisms (porosity %, for example). Based on three different weightings, first, the dried sample was weighted (m1), after that, the sample is weighted after eliminating the air, using a desiccator, and saturated in a water tank for 72 h: denoted (m2). Third, the saturated sample is wiped superficially to remove surface water: denoted (m3). Finally, the sample total porosity is given as follow:

1 $$Porosity = \, \left( {\frac{{m_{3} - m_{1} }}{{m_{3} - m_{2} }}} \right) \times 100$$

**Figure 3:**
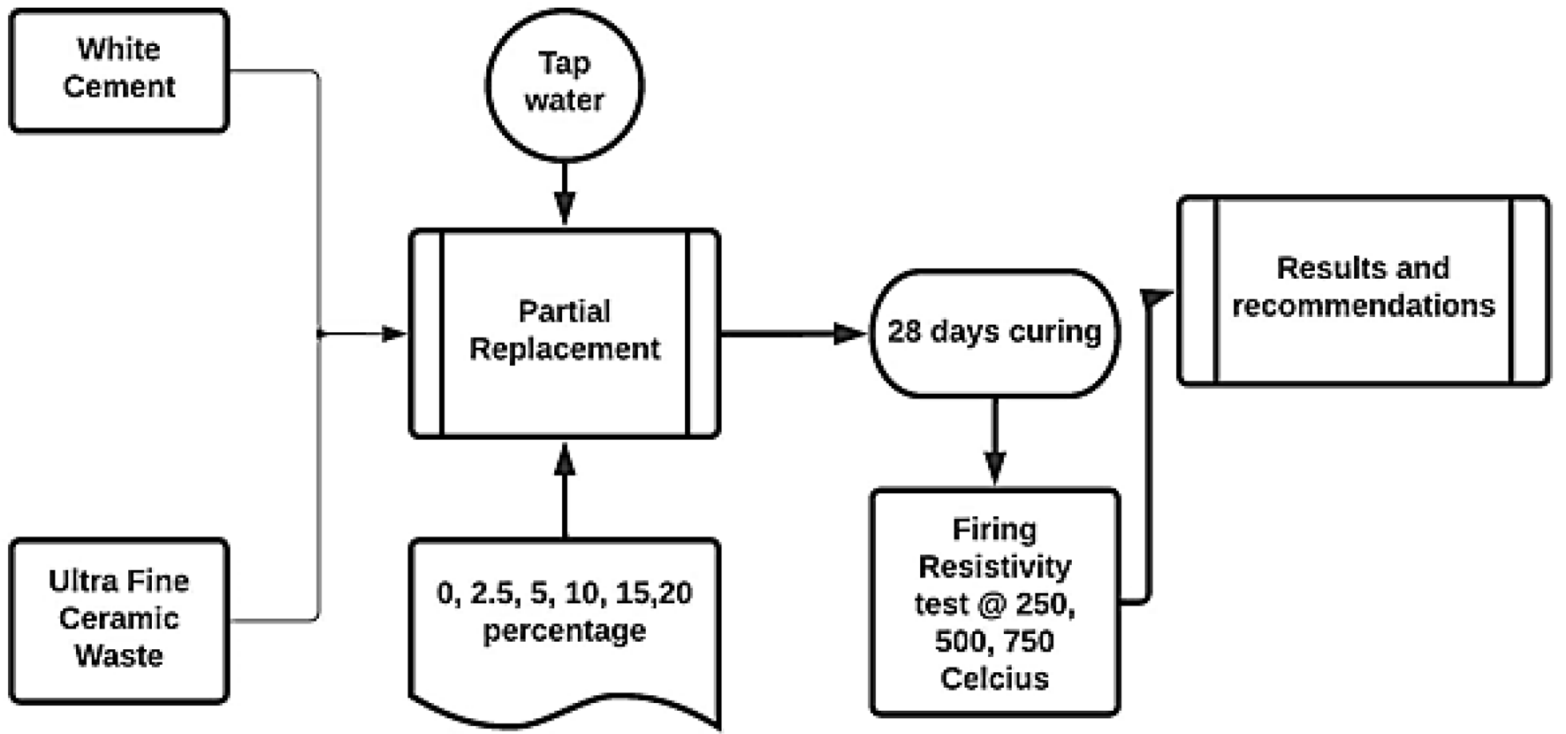
Methodology flow chart indicating the research method.

Pore diameters were recorded and characterized, with a baseline established for each (macro-pores larger than 3500 nm, micro-pores in 0–15 nm, and meso-pores from 15 till 3500 nm)^35^. The small pieces from the burnt specimen was saved for later X-ray, infrared, and scanning electron microscopy (SEM); examination. In Fig. 3, we can see the experimental plan's scientific outline.

### Instrumental Analysis

Specimen morphology conducted with (FEI Company, Netherlands) integrated with EDXA namely, “*an energy dissipation X-ray analyzer*”. Other fired samples were pulverized, stored, dried, and passed from 25 µm mesh to determine the hydration phases after firing using X-ray diffraction (XRD, Philips PW3050/60) diffracto-meter order; 5 and 50 (2Ø), speed rate of 1 s step^−1^ and high resolution accuracy of 0.05° step^−1^^36^. The transmission electron ultra-microscopy (TEM) instrument reported that the effective particle size for CW is 100–200 µm. This indicated that, CW in micro-size powder, as shown in Fig. 1b, which a suitable particle size form meso-pores of the WC blends.

## Results and discussion

### Phases of hydration

#### XRD-patterns

**Figure 4:**
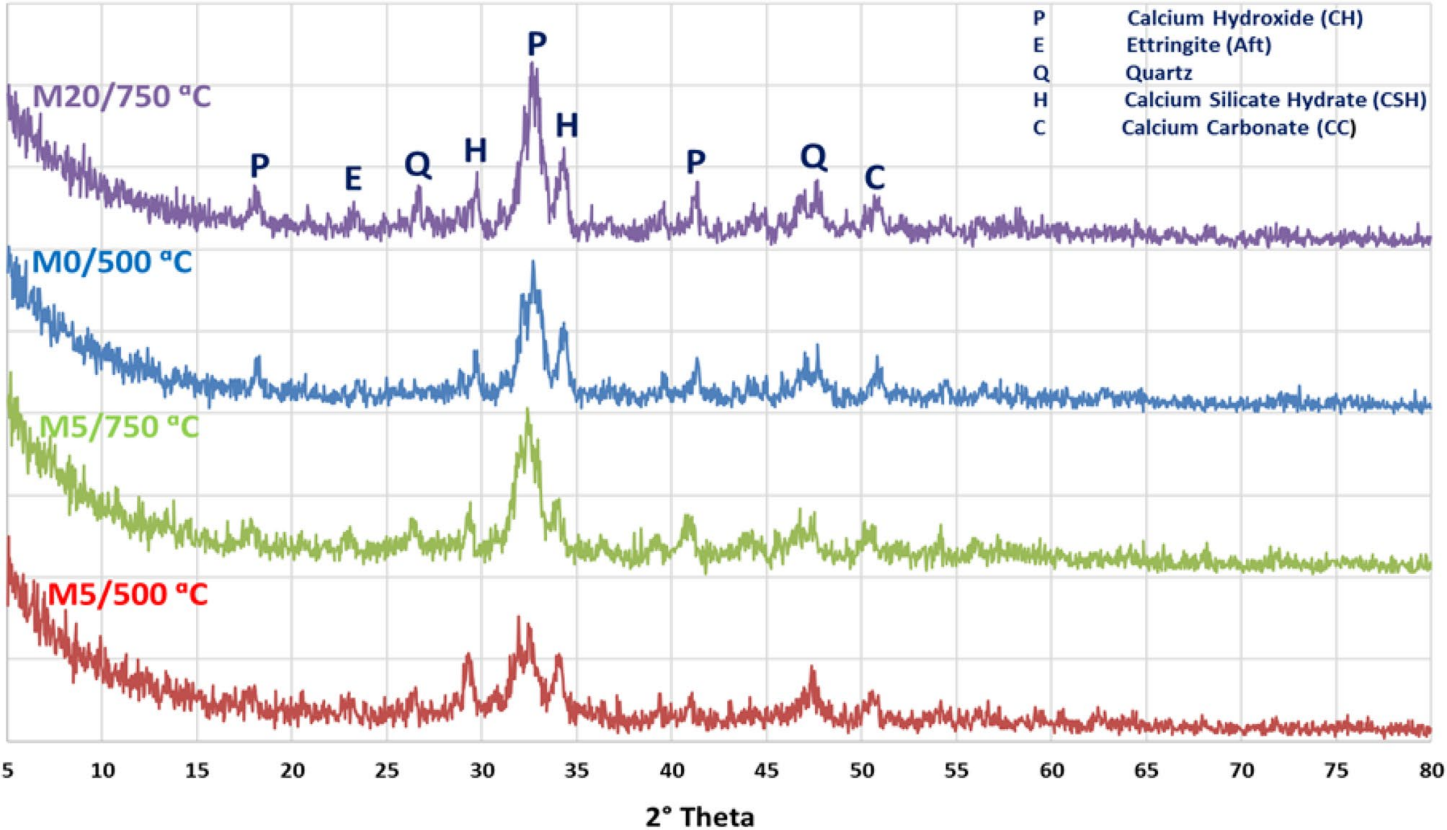
XRD pattern of hardened M5/500 °C, M5/750 °C, M0/500 °C and M20/750 °C pastes after 28 days of hydration then exposed to different thermal temperatures.

**Figure 5:**
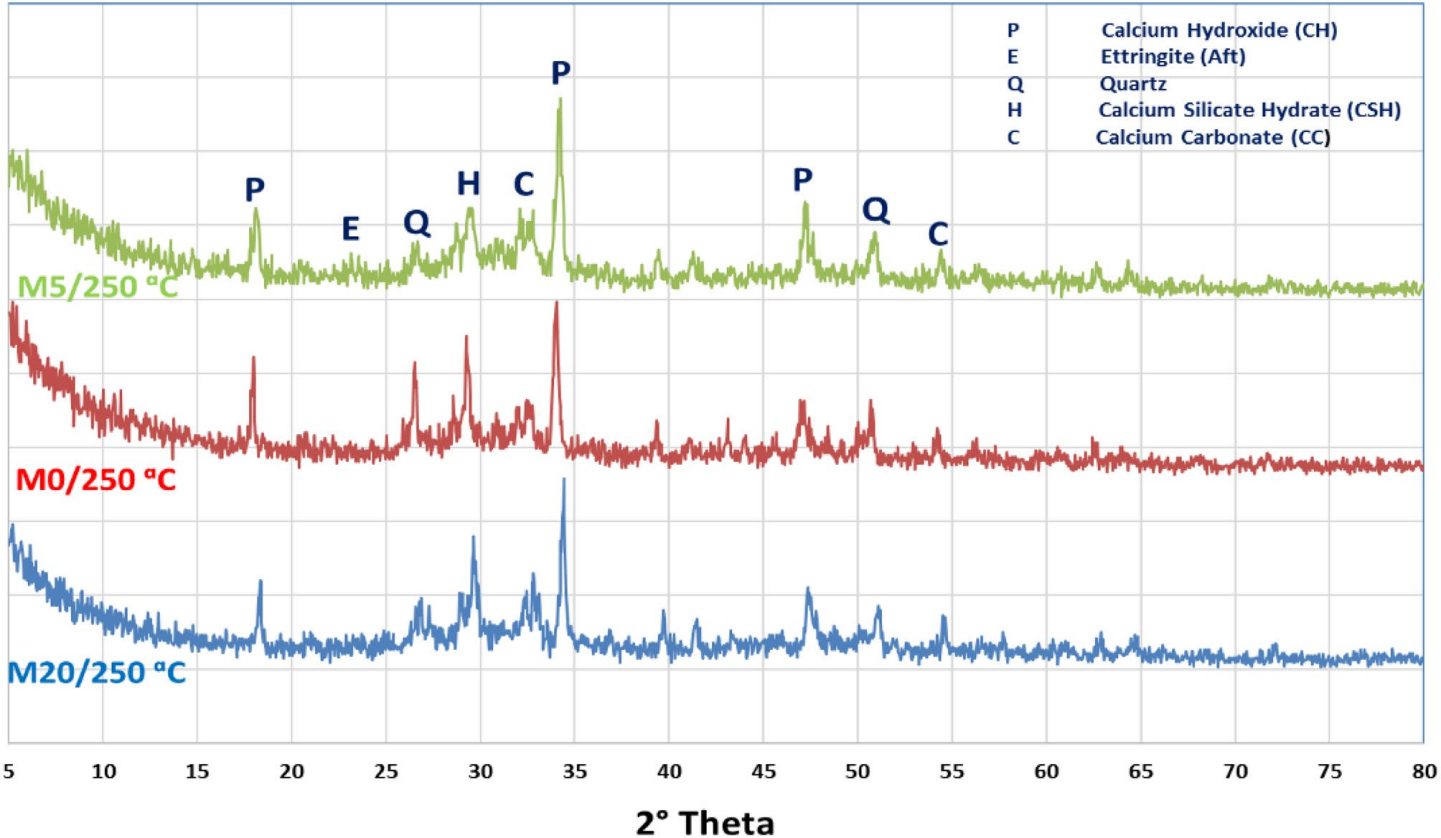
XRD patterns of hardened M0, M5 and M20 pastes after 28 days of hydration at 250 °C.

Composites WC-pastes with CW powder hydrated for 28 days, then excesses to elevated temp. up to 750 °C, the phases of curing showed in Figs. 4 and 5, respectively. By using XRD-patterns the number of hydration, products before and after firing were evaluated. The X-ray diffractograms beaks showed that the hydration phases mainly depended on the amount of CW content especially late ages of hydration^37^. Firstly, thee XRD-patterns of M5/500 °C, M5/750 °C, M0/500 °C and M20/750 °C plotted in Fig. 4. Clearly, the main hydration products responsible for compressive strength and bulk density such, as C–S–H and calcium hydroxide (C–H), were observed in all mixes with different ratios. Which responsible about the compressive strength and increase in the bulk density for both M0 and M5 at different thermal temperatures^38^. Due to the high replacement effect M20/750 °C caused low strength with the high total porosity. This may be attributed to the presence of high quartz content (un-reacted silica) appearing in the XRD patterns. The mixes containing 5.0% wt% of WCs fired at (M5/500 °C and M5/750 °C) presented highly C–S–H phase intensity comparing with anther mixes (M0/500 °C and M20/750 °C). This confirmed the results of compressive mechanical strength and high firing resistivity of the M5 blend.

Figure 5 shows the XRD patterns of hardened M0/250 °C, M5/250 °C and M20/250 °C pastes after 28 days of hydration. This proves that M5/250 °C shows higher workability and synergist properties verses M0/250 °C and M10-20/250 °C mixes. It can be attributed to; M5 has the maximum fill index, then other composites where further replacement has lower hydraulic impact and poor fire resistance. This might explain the high intensity peak of both C–S–H along C–A–H, in the case of M5/250 °C-mix. Additionally, M20/250 °C-mix possess low synergetic features as the dilution effect. This dilution led to high porosity with low firing resistivity. In the following order M5/250 °C > M5/500 °C > M5/750 °C > M20/250 °C ˃ M0/250 °C ˃ M20/750 °C ˃ M0/500 °C ˃ M0/750 °C, represent the synergic resistance features which is confirmed by other works too^39^.

#### FTIR spectra

**Figure 6:**
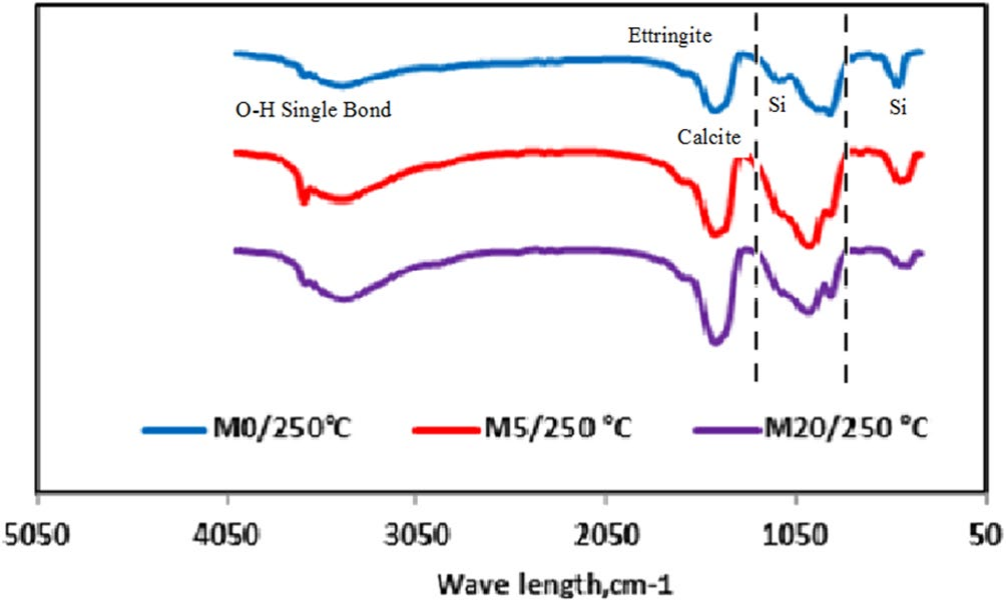
FTIR spectra of hardened M0, M5 and M20 pastes after 28 days of hydration at 250 °C.

Figure 6 shows the results of FTIR analysis performed on mixtures of M0/250 °C, M5/250 °C, and M20/250 °C. The symmetric and asymmetric O–H single bond found in water, yielding calcium hydroxide (C–H), is the source of the band at 3641 cm^−1^. After being heated to 250 °C, the spectral absorption band at 1650 cm^−1^ in M0 was found to be related with ettringite. While the absorbance band vanished after being heated, the XRD data were consistent with the breakup of ettringite at high temperatures, demonstrating its thermal instability. Silica absorption bands emerge at 469 cm^−1^, 785 cm^−1^ (present in the non-hydrated cement), and 1052 cm^−1^ (present in the hydrated cement)^40^, which is an intriguing finding illustrated by the two dotted lines. The polymerization of SiO_2_ four units in Alite (C3S) and Belite (C2S) was responsible for this finding. M5F has the greatest and broadest absorption at 1050 cm^−1^. This is consistent with the results of the compressive strength tests, and it may be attributable to the addition of the right amount of CW, which resulted in greater compressive strength compared to the others. As can be seen in M20F, the addition of additional CW decreased the compressive strength because of the presence of too much unreacted SiO_2_.

**Figure 7:**
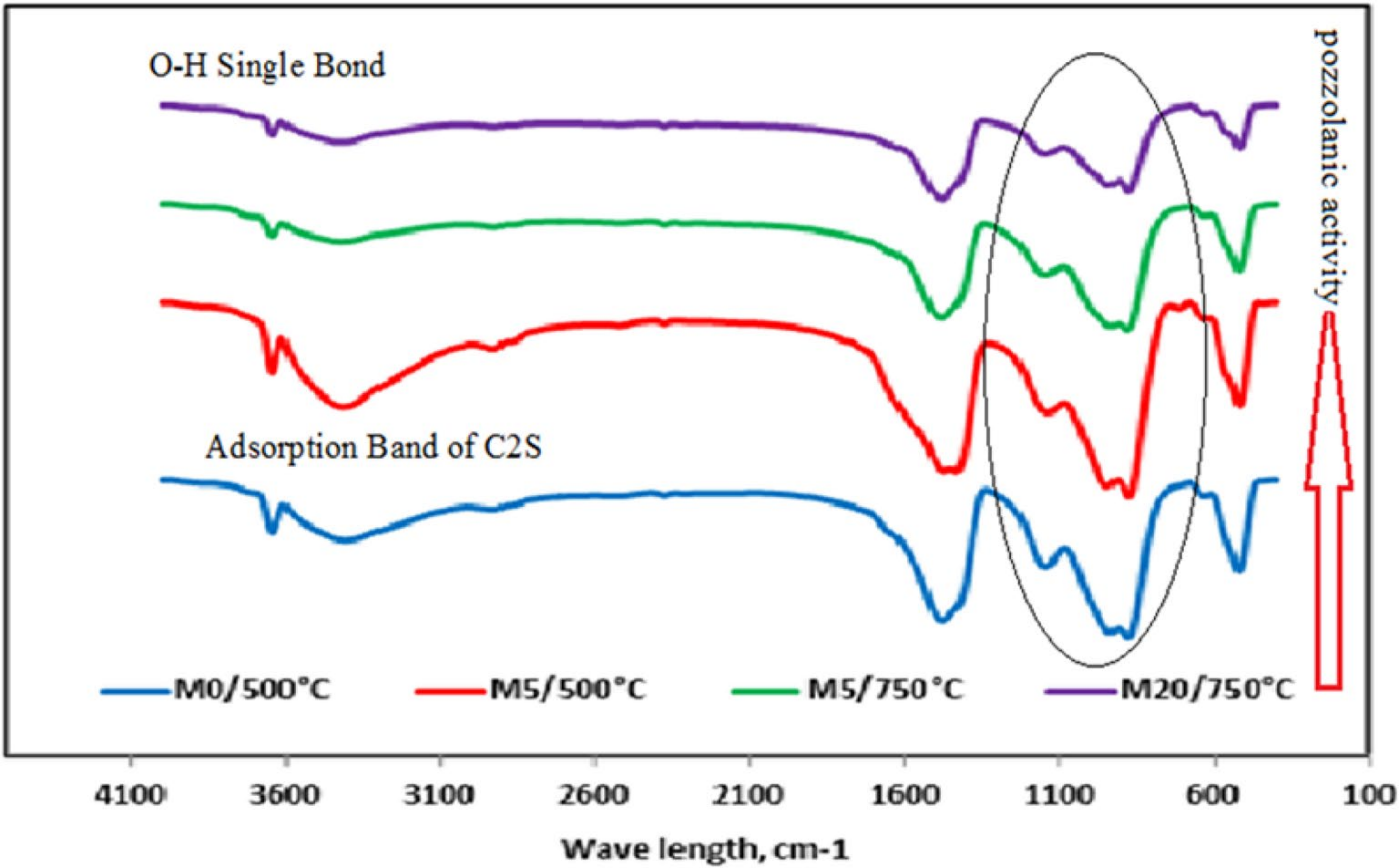
FTIR spectra of hardened M0, M5 and M20 pastes after 28 days of hydration at different thermal temperatures.

Comparing the spectra of control sample M0 with M5F at incarnation temperature 250 °C and 750 °C respectively, it is evident that a portion of C–H was re-structured to carbonate and the decomposition above 750 °C, band intensity decreased^41^. Finally, the intensity of C2S band was increased by raising the temperature and vice-versa. The FTIR spectra of M5F as shown in Fig. 7 demonstrated a similar evolution as those of M0. Compared to the FTIR spectra of M5F, the peaks related to the vibration of O single bond ^−^H in C–H at 3641 cm^−1^ cannot be observed in all FTIR spectra of M0. This demonstrates that C–H is used up in the pozzolanic process and that CW's pozzolanic activity is activated by heat. Eventually, ceramic waste replacement up to 5.0 wt% is suitable and reflects improved performance and energy saving qualities, lead to reduced construction costs and higher sustainability of raw materials.

### Physico-mechanical features

#### Compressive mechanical strength (CMS)

**Figure 8:**
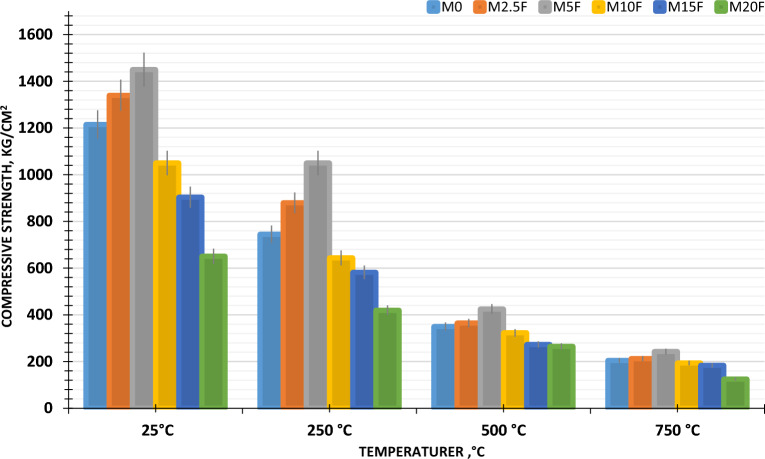
Compressive strength of blended WC-pastes with or without CW for 28 days of hydration at different thermal temperatures.

Results of CM’s verses during periods for WC-pastes incorporated with ultrafine-CW at different thermal temperatures are depicted in Fig. 8. As shown in figure, the CM’s of specimens blended with or without ultrafine-CW at various thermal temperatures increased with increasing hydration age. For hardened prism still 5% (M5F) replacement at 25 °C, the blend's compressive mechanical strength increases with hydration progress yielding extra products, leading of extra species of hydration products, particularly C–S–H, which is responsible for solidification features^42^. When tested against other mixtures and the neat sample (M0), the hardened paste M5F had the highest mechanical compressive strength. It might be concluded to the Pozzolanic features of CW during the late age of the hydration process, consuming high content of Ca(OH)_2_ accompanied by extra production of C–S–H gel, where it was observed as (honeycomb-like) on WC-surface and pores as the nucleation effect leads to densification in microstructure^43^.

#### Compressive strength fluctuation

**Figure 9:**
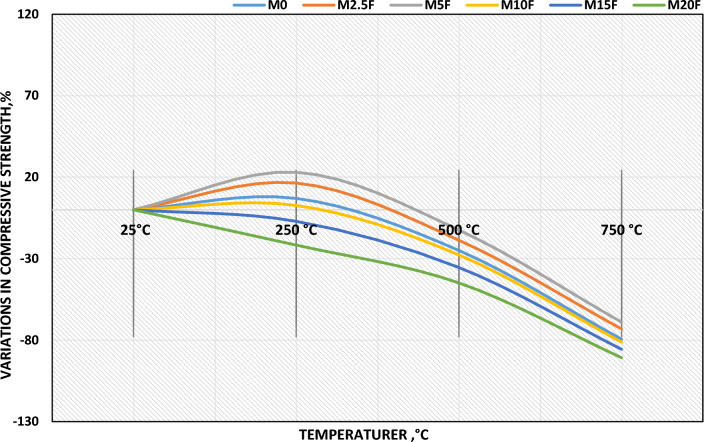
Variation in compressive strength of blended WC-pastes with or without CW for 28 days of hydration at different thermal temperatures.

After 28 days of hydration compressive strength variation is obtained as mentioned. Figure 9 shows the percentage of CS; fluctuation of WC-pastes incorporated with CW when subjected to increased temperatures up to 750 °C. For all WC-pastes, compressive strength decreases dramatically at temperatures above 750 °C after initially increasing after thermal load to 250 °C. At thermally load up to 750 °C, M5F has been shown to have the highest MCS; variations and the higher resistance to fire compared to other tidy MF's. This is because the water steam inside the pores of WC-pastes eliminates the physically adsorbed water, resulting in a self-autoclaving effect that speeds up the hydration process of the un-hydrated WC-clinker. Clearly, all WC-pastes benefited from the tested temperature range of 0–250 °C. In addition, self-autoclaving WC-pastes increases the polymerization degree of C–S–H^41,44^. At temperatures as high as 750 °C, compressive strength significantly decreased. This is mostly because the strength-giving hydration stages have broken down. In contrast, M5F had the maximum compressive strength at both 500 and 750 °C during firing, and it was the strongest overall. This is because an excessive amount of hydration products formed, which countered the firing effect. When compared to the M5F-mix, all of the WC-pastes in which 2, 5, 10, 15, and 20 weight percent of CW was substituted had the same syndrome: low fire resistance across the board. This is attributed to the increment of WC-pastes porosity due to the highest coarsening of MF's pore size distribution and decomposition of hydrated phases, formed an amorphous Portlandite (poor crystalline structure) and Gehlenite (Ca_2_Al_2_SiO_7_); at thermal load up to 750 °C. However, the M20F mix presented the lowest compressive strength regression especially, at 500 °C and 750 °C. This is explained the dilution of WC-matrix with low hydration products. It can be proven that, each hydration product and the compact are central to the variations in compressive strength under firing temperatures^45^.

#### Total porosity (TP)

**Figure 10:**
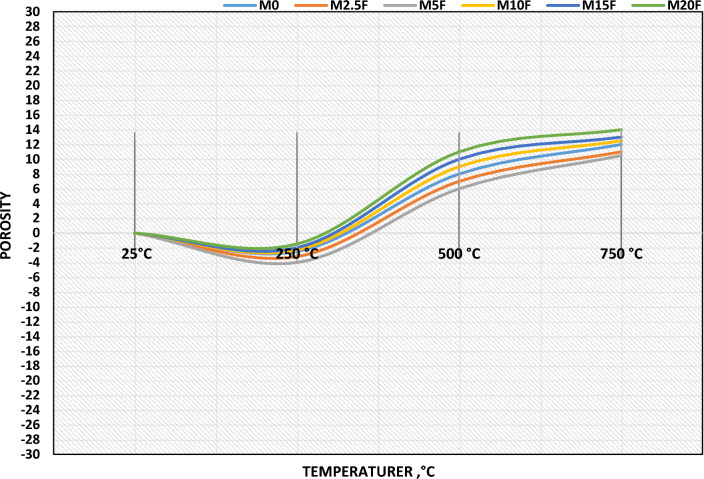
Porosity of blended WC-pastes with or without CW of 28 days of hydration at different thermal temperatures

After 28 days of hydration and exposure to increased temperatures up to 750 °C change in porosity is calculated. Figure 10 displays the differences in porosity between blended WC-pastes with and without CW. Up to 250 °C, it was evident that the porosity of all WC-pastes (with or without CW) was slightly reduced. Self-autoclaving WC-paste is a process that reduces the number of micro-pores in the material's matrix. On the other hand, for all WC-pastes, porosity differences greatly increased after 250 °C, especially at 750 °C. The expansion and proliferation of microscopic holes and fissures is to blame. An increase in the crystallinity degree (CD) of hydration products (HP) occurs at higher temperatures, and this is manifested as the formation of open pores in the WC-matrix^46^.

In comparison to the other mixtures, the WC-paste with 5.0 wt% CW (M5F) showed the least amount of porosity across the board. The impact of CW, which closes down pores and reduces micro-cracks, is responsible for this. These results tied in with the ones for compressive strength and demonstrated the universal link between the two variables. When comparing M20F and M15F pastes, it was clear that a different pattern emerged, with the M20F sample displaying the maximum porosity up to 750 °C. These results shed light on why CW affects the permeability of WC-pastes. Lower porosity WC-pastes had greater firing resistance and compressive strength. These findings validated the probity and firing resistance of CW, which were a reflection of its filling and nucleating capabilities^47,48^.

#### Weight loss

**Figure 11:**
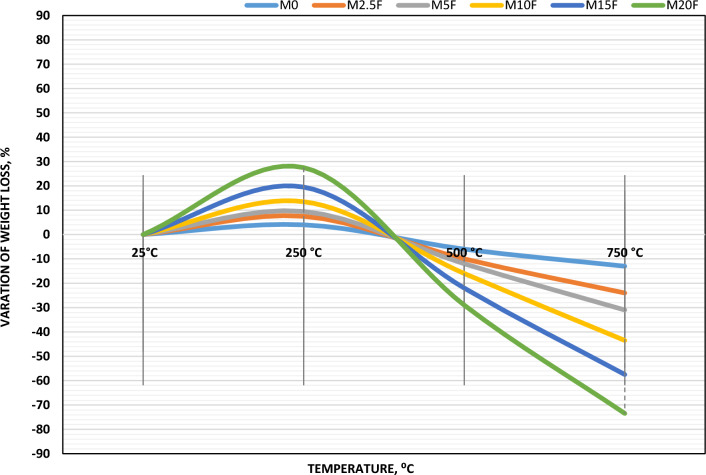
Variation of weight loss of blended WC-pastes with or without CW of 28 days of hydration at different thermal temperatures.

Weight loss identification is an important parameter to be noted while adding any supplementary materials. Figure 11 represents a visual illustration of the percentage of weight changes of WC-pastes incorporated with CW and exposure to increased temperatures up to 750 °C. Notably, the weight loss variation for all pastes dropped up to 500 °C, then increased up to 750 °C, as the temperature was increased. This is accompanied by the fire effect, which causes the disintegration of hydration products and the loss of free water (FW) at 100 °C and combined water (CPW) up to 600 °C. At 250 and 500 °C, all WC-pastes showed the lowest weight losses regardless of whether or not they contained CW. This points to a drop in FW and CPW proportions. Weight loss was greatest for the M20F mixture, despite its high CW content, and lowest for the M5F sample. This lends credence to the idea that CW-filling causes an excessive amount of hydration products (HP) to be present in the WC-matrix. This forms C–S–H and Ca (OH)_2_ which decompose at around (300–400 °C). According to instrumental analysis in the previous figures, CaCO_3_ was formed as a hydration product resulting from CW-hydration and normal carbonation^49–51^. This product begins to decomposition at 600 °C. This confirms the increment in weight loss at 750 °C in case of M20F comparing with M5F. The weight losses of WC-Pastes containing CW except M20F are lower than this paste (M20F) and the control sample (M0). especially at 500 °C. This may be attributed due to the presence of additional hydration products (C–S–H and C–H) in WC-matrix, which are de-hydroxylated at range (300–400 °C)^52^.

#### Whiteness reflection (Ry)

**Figure 12:**
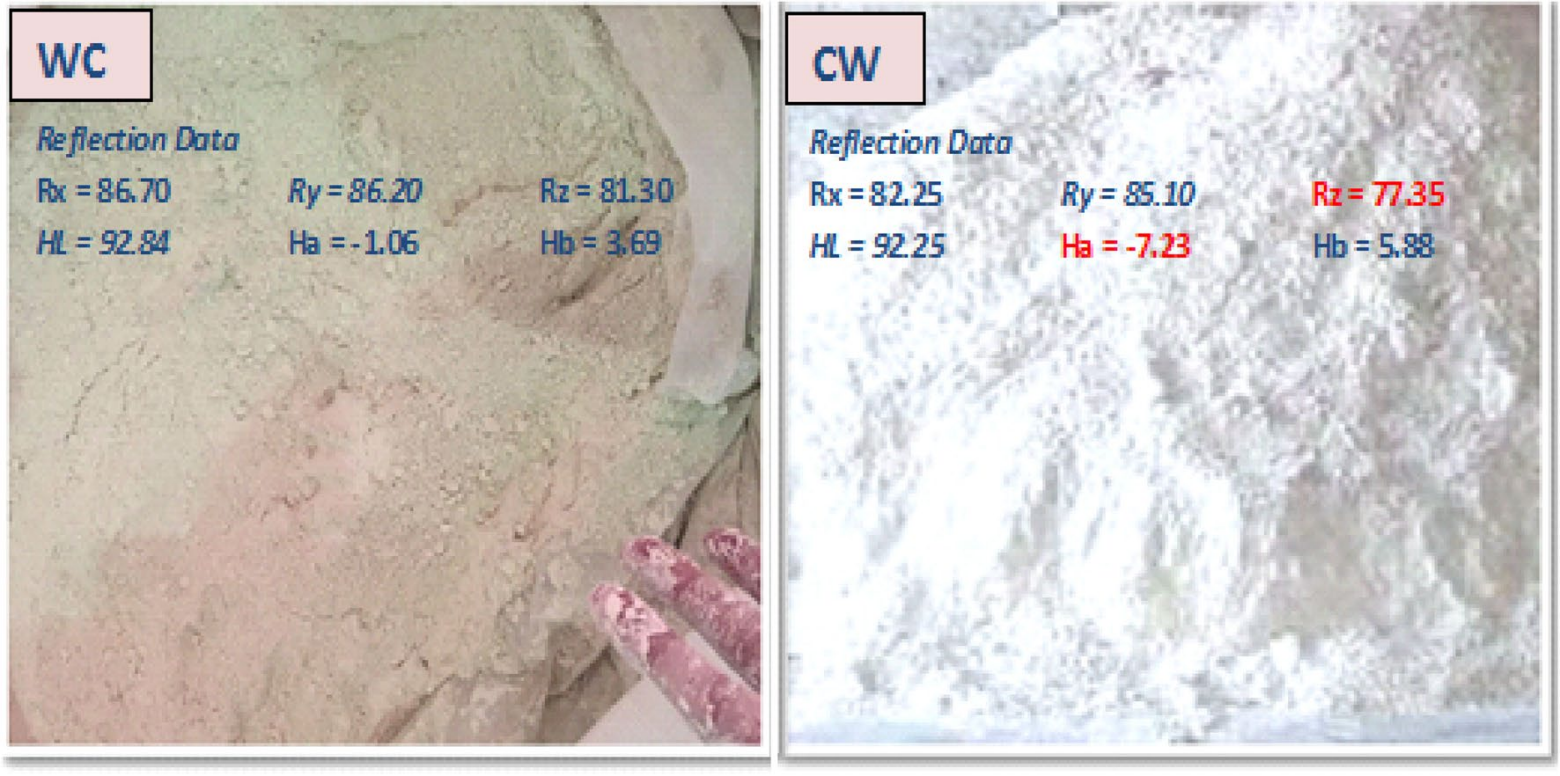
Visual comparison between WC vs. CW.

**Figure 13:**
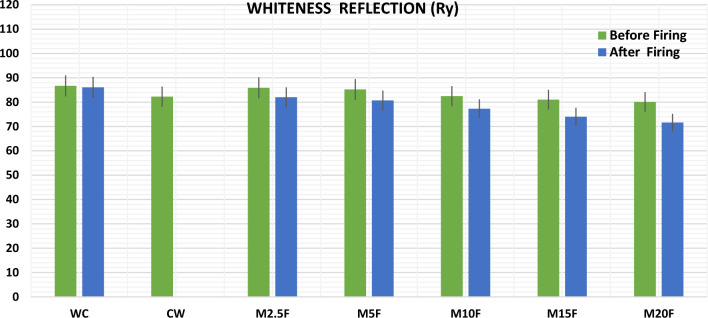
Ry for pastes before firing and after firing on dray base.

While CW appeared white after milling, its reflectance using the "Elerpho" apparatus was inadequate, normally on the Rz-axis that causes green color reflection, as shown visually in Fig. 12: Ha = − 7.23 (very pale yellow) versus Ha = − 1.06 (very crimson green white). This solid proof supports the role of consumption in the reversal of the whiteness reflection of the blends (Ry). Table 4 and Fig. 13 show the findings of measuring the Ry; of WC incorporate with ultra-fine CW powder.

**Table 4:** Ry for pastes on dray base.

Blend	WC	CW	M2.5F	M5F	M10F	M15F	M20F
Ry							
Before firing	86.70	82.25	85.90	85.20	82.45	81.03	80.11
After firing	86.10		82.00	80.70	77.30	74.00	71.60

Elerpho reflected that the whiteness reflection values were decreasing with an increasing in the CW powder content. It can be summarized as: Ry-M0 > Ry-M2.5F > Ry-M5F > Ry-M10F > Ry-M15F > Ry-M20F, conform to DIN 5033 (relative to 100% Rx, Ry and Rz)^53^. The Ry; values vary from 86.70% of (M0) for WC without CW-powder to 80.11% of (M20F) with increasing the content of CW-powder replacement. Moreover, the whiteness level of (M2.5F) reported a better green reflection on Rz-axis value by 85.90%. This can be attributed to, the negative green reflection and positive yellow reflection of CW-powder, which equal to 82.25%, may marginally affect the whiteness level of WC-blends. Frankly, CW can be proposed as a possible additive for the production of white cement up to 5%, without any negative defects at the level of whiteness of the final product^54–56^. After 28 days of curing blends have been fired at different thermal temperatures. Small species were collected to check the whiteness reflection level; it was noticed that all blends have affected negatively after firing. Ry of WC shows the best performance, i.e.: Ry slightly moved from 86.70 to 86.10. In the contrary; others Ry results decrease by 4.09, 5.30, 6.24, 8.67 and 10.62 respectively for M2.5F to M20F. XRF analysis of CW shows high content of ferric oxide higher than WC content by 92.70%, which may have a stronger effect on Ry reduction of blends after fire, ferric turns into ferrous, i.e.: turns pale yellow orange-red when heat treated.

#### Morphology and microstructure

**Figure 14:**
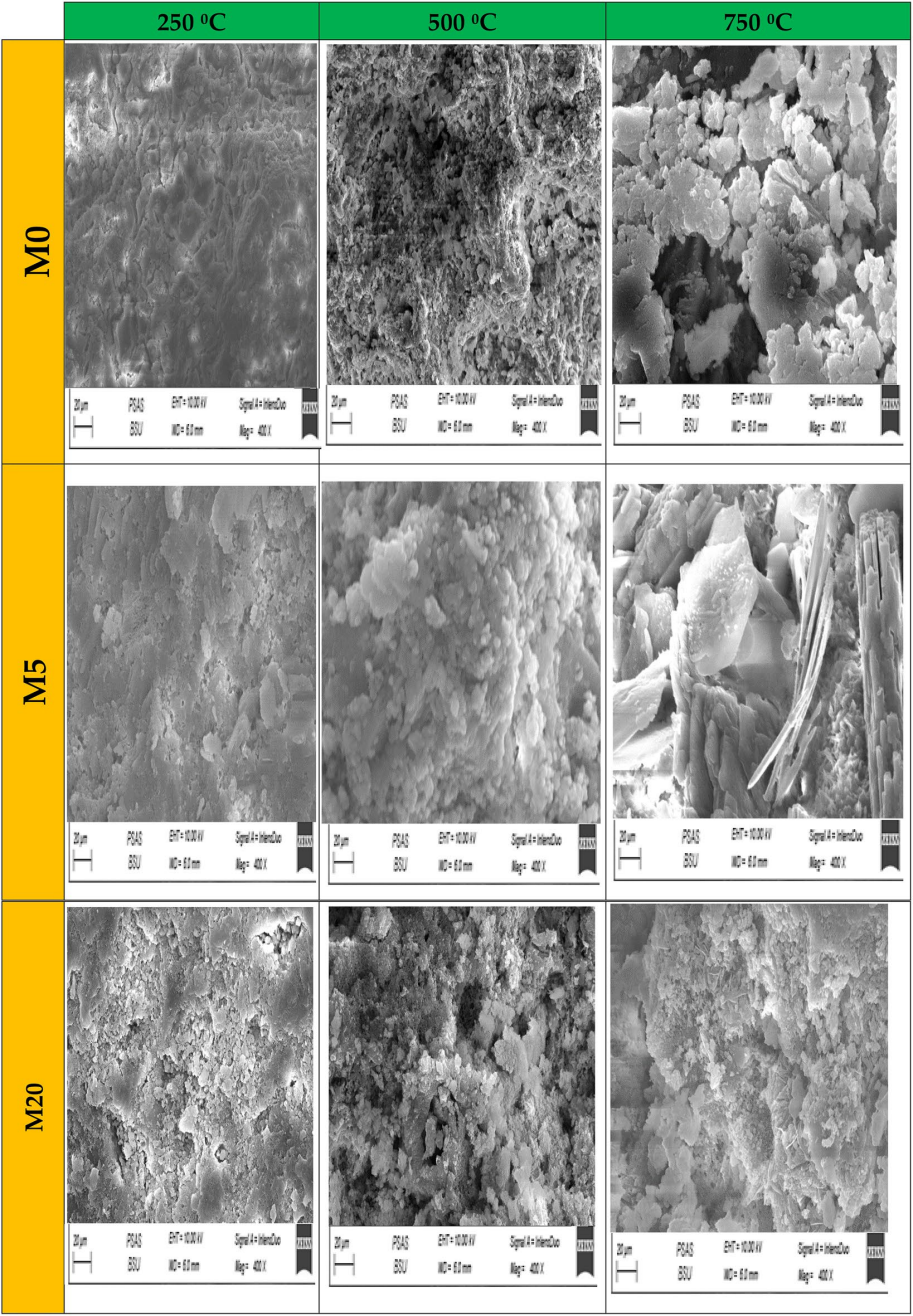
SEM-photos of hardened M0, M5wc-F and M20wc-F pastes after 28 days of hydration at different 250 °C, 500 °C and 750 °C.

The microstructure variations during thermal treatment from 250 °C up to 750 °C, were examined by SEM-photos as shown Fig. 14. According to compressive strength results, 500 °C is regarded as a critical firing temperature, that determines the dual effect of nucleating and Wt., percent CW on the performance of WC-pastes under elevated temperatures. It can be shown that more densification in the microstructure in the case of firing at 250 °C reflected a significant effect on the performance of WC-pastes under elevated temperatures^57–59^. The SEM-photos proved the higher compaction of M5F at 250 °C microstructure compared with M0 and M20F at the same temperature, confirming the positive impact of CW on the microstructure densification as well as the performance under elevated temperatures. The SEM micrograph of the paste made from neat WC at 250 °C reveals the formation of well-developed hydration products such as calcium hydroxide (C–H) crystals intermixed with small wrinkled fibres of calcium silicate hydrate (C–S–H); and calcium sulphoaluminate hydrate (Ettringite). Besides, the pore spaces are still available for depositing new hydration products^60^. In the same direction, the morphology of M5F fired at 500 °C found to be denser with more compact than M0 and M20F at the same temperature. This confirms the fact that, the composition of cementitious materials as binder represents as good impact on the firing resistivity. Although CW has a good effect on microstructure densification due to its increased nucleating sites effect, the microstructure of all mixes at 750 °C appears to be less compact than the identical mixes at 250 °C and 500 °C. At fire temperatures, the M5F-mix outperformed other mixes in terms of microstructure. The results of the compressive strength, porosity, and weight change fluctuation are all in good agreement with the SEM-morphology.

## Conclusion

The improvement of solid waste recycling to produce eco-cement is a goal of the sustainability aspects which is practiced by many researchers. One of the way to use waste materials is to impregnate them in productive materials such as concrete, building materials etc. Huge quantities of ceramic waste produced as byproduct of industries need to be utilized and it need further research to maximize its benefits. This work represents one such solution of reuse of the CW as a complementary raw material in white cement production without a negative impact on its whiteness reflection (Ry) degree. Five suggested patches of cw were studies, physico-mechanical features and microstructure investigations were studied to understand the impact of adding ceramic waste into white cement. Combination M5F reported increases in mechanical strength, whiteness reflection (Ry), setting and synergetic properties compared to others mixes. WC replaced by 2.5, 10, 15 and 20 with a weight of CW shows a similar syndrome; poor thermal resistivity, which may have attributed to increases in paste porosity and the decomposition of hydrated phases form of amorphous Portlandite (poor crystalline structure) and Gehlenite (Ca_2_Al_2_SiO_7_); at thermal load up to 750 °C leads to crack formation and decreases of its strength values. It recommended that 5.0% of CW is applicable with additional physic-mechanical features. This work provides information related to the dosage and method of utilizing ceramic waste into cement production.

## Data Availability

The datasets used and/or analysed during the current study available from the corresponding author on reasonable request.

## Abbreviations

*WC*: White cement
*CW*: Ceramic waste
*SCMs*: Supplemental cementitious materials
*Ry*: Whiteness reflection
*HL*: Hinter L (whiteness intensity)
*Ha*: Hinter a (green color intensity)
*Hb*: Hinter b (yellow color intensity)
*MCS*: Mechanical compressive strength
*W/CP*: Water/cement powder ratio
*C-S-H*: Tobermorite gel
*ST*: Setting time
*CD*: Crystallinity degree
*TP*: Total porosity
